# Gastric Diverticulum Presenting as Hematemesis: A Case Report Detailing an Uncommon Presentation of an Already Rare Entity

**DOI:** 10.5811/cpcem.2020.9.48603

**Published:** 2020-10-26

**Authors:** Maddi Massa, Karla Newbold

**Affiliations:** Spectrum Health Lakeland, Department of Emergency, Saint Joseph, Michigan

**Keywords:** Gastric diverticulum, hematemesis

## Abstract

**Introduction:**

Gastric diverticula (GD) are uncommon. Most are asymptomatic and diagnosed incidentally. Symptoms range from reflux and epigastric discomfort to life-threatening bleeding and perforation. We describe a case of symptomatic GD presenting as hematemesis requiring surgical treatment.

**Case Report:**

A 57-year-old female presented to the emergency department (ED) with one day of epigastric pain and hematemesis. Hemoglobin was found to be stable, but blood urea nitrogen was elevated. Imaging revealed a fundal GD. Esophagogastroduodenoscopy did not show other etiology of hematemesis. The patient underwent partial gastric resection for GD removal and did well without further symptoms on follow-up.

**Conclusion:**

Although rare, GD needs to be included on a differential diagnosis when evaluating gastrointestinal symptoms in the ED. Patients may present with an array of complaints but can potentially develop serious complications. Providers should be familiar with the diagnostic options and treatment regimens available to better care for patients presenting with GD.

## INTRODUCTION

Gastric diverticula (GD) are extremely rare.^1^ Prevalence ranges from 0.04% on imaging studies and 0.01–0.11% on esophagogastroduodenoscopy (EGD).^2^ Most diverticula are diagnosed incidentally, as the majority are asymptomatic.^2^ Symptomatic patients can experience a wide range of ailments from mild discomfort and reflux to perforation and life-threatening bleeding.^3^ Management depends on symptoms and diverticular size.^3^ Although most patients with GD are diagnosed on an outpatient basis, or other subsequent workup, this case report demonstrates an emergency department (ED) diagnosis of a bleeding GD requiring surgical treatment.

## CASE REPORT

A 57-year-old female presented to the ED with upper abdominal pain and hematemesis for two to three hours. She had been experiencing malaise and fatigue during the preceding night. Past medical history included schizoaffective disorder, diabetes, gastroesophageal reflux disease (not currently on medical management), and chronic pain. She lived at an adult foster-care facility. Due to her underlying psychiatric issues, history was very limited. She reported associated nausea with vomiting three times. These episodes involved bright red blood about the size of her palm. She reported normal bowel movements. She did smoke cigarettes but denied any alcohol or illicit drug use.

Vital signs revealed slight tachycardia with heart rate at 105 beats per minute. Other vital signs were within normal limits with blood pressure of 101/65 millimeters of mercury, temperature of 97.9 degrees Fahrenheit, and respirations of 20 breaths per minute with 94% oxygenation. On physical examination, she was in mild distress from pain. The patient exhibited tenderness to palpation of the epigastrium, as well as the right and left upper quadrants, without rebound or guarding. Laboratory analysis revealed results significant for a hemoglobin of 14.1 grams per deciliter (g/dL) (reference range 12–16 g/dL), a blood urea nitrogen of 24 milligrams (mg)/dL (reference range 6–20 mg/dL), normal prothrombin time, normal partial thromboplastin time, and normal platelets. An upright chest radiograph did not reveal free air under the diaphragm or other signs of perforation. The patient was initially treated with normal saline bolus, anti-emetics, pantoprazole, and fentanyl.

**Image 1:**
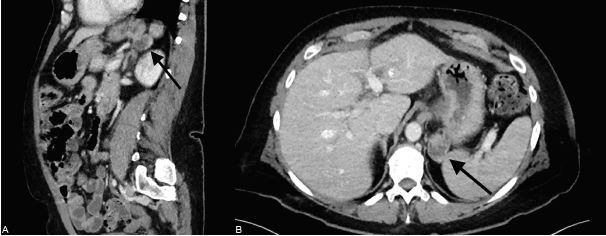
Sagittal (A) and axial (B) images illustrating a 3.1-centimeter posterior-inferior fundal gastric diverticulum on computed tomography using intravenous contrast.

**Image 2:**
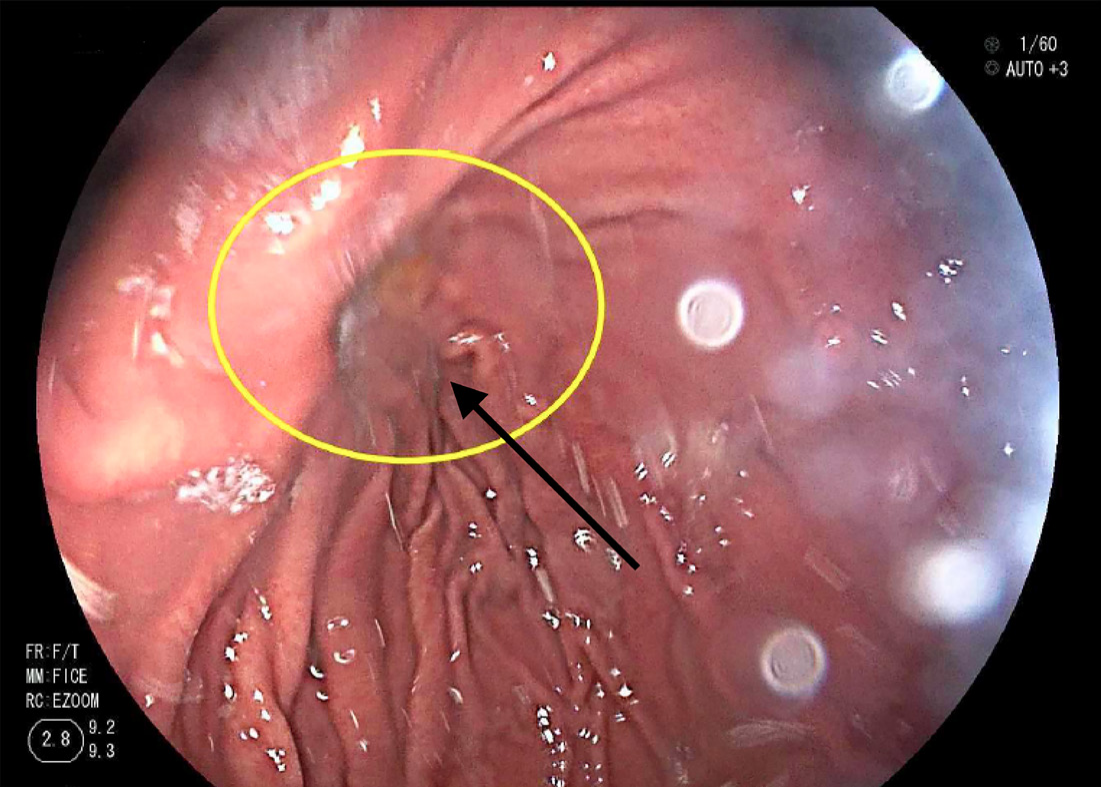
Esophagogastroduodenoscopy showing the mouth of a posterior-inferior fundal gastric diverticulum.

Due to limited history, a computed tomography (CT) of the abdomen and pelvis was also ordered. CT of the abdomen and pelvis revealed a 3.1-centimeter (cm) posterior-inferior fundal gastric diverticulum. (Image 1). General surgery consult recommended pantoprazole infusion and planned for EGD later that day. The patient required additional pain management. She underwent EGD the next day and was found to have a wide- mouthed gastric diverticulum at the posterior fundus without active signs of bleeding. (Image 2). No other abnormalities on EGD were noted. The procedure had to be aborted slightly early due to hypoxia that resolved with simple maneuvers.

She did not have any further pain, nausea, or hematemesis during the remainder of her hospitalization. She was discharged the next day on proton pump inhibitor (PPI) therapy with plans for further partial gastrectomy and diverticulectomy. She returned one week later and underwent a laparoscopic resection of the gastric diverticulum and lysis of adhesions. She had an unremarkable postoperative course. Upon follow-up 11 days later, she had no further issues and has not returned with recurrent symptoms. She was continued on PPI therapy. Pathology of the diverticulum revealed normal gastric mucosa without malignancy.

CPC-EM Capsule

What do we already know about this clinical entity?

*Gastric diverticuli (GD) are rare and usually diagnosed incidentally or outpatient. Most are asymptomatic but severe complications can occur*.

What makes this presentation of disease reportable?

*This case was diagnosed from the emergency department (ED) with excellent computed tomography images. This author was unable to find any GD reports in ED literature*.

What is the major learning point?

*Emergency physicians need to keep this entity on their differential as perforation, significant bleeding, malignancy, and infectious sequalae can all result from GD*.

How might this improve emergency medicine practice?

*Increased awareness of GD, and management options, should improve patient centered care with both severe and mild cases of GD*.

## DISCUSSION

Gastric diverticula are extremely uncommon, being the least common type of intestinal diverticula.^1^ They tend to affect males and females equally.^1^ Normal diverticular size is usually between 1–5 cm, but sizes up to 11 cm have been described.^1^,^3^ Classification can be subdivided into true (congenital) and false (acquired) diverticula; with true diverticula involving all layers of the gastric wall.^3^ False diverticula can be further classified into pulsion and traction diverticula, which result from increased intraluminal pressures or perigastric adhesions.^3^ True diverticula are more common (70–75% of GD) and arise from the posterior fundus, whereas false diverticula are usually found at the antrum of the stomach.^1^,^3^

The favored means of diagnosis is with EGD or upper gastrointestinal (GI) contrast radiographic study. While CT can be used for diagnosis it is not as reliable as the aforementioned methods due to the risk of misdiagnosis; however, oral contrast included in CT protocol may help increase sensitivity.^2^

Management of gastric diverticula is based on symptoms. Most asymptomatic patients do not require any treatment.^2^ Patients who experience reflux, epigastric pain, and halitosis can be trialed on a PPI. This may not be effective, however, as it does not address the underlying pathophysiology of the GD; food and gastric juices can still be retained in the diverticulum, causing distention, pain, and subsequent reflux.^3^ It is thought that diverticula with larger mouths are less symptomatic due to decreased chance of content retention.^1^,^3^ Some portion of patients with diverticula have other GI pathology. GD may simply worsen symptoms or conversely may not affect them at all.^1^ Surgical management is preferred when diverticula are large, symptomatic, cause bleeding, perforation, or are associated with abscess and malignancy.^2^ Laparoscopic resection, possibly with intraoperative EGD, is preferred over laparotomy.^1^

## CONCLUSION

Due to the potentially life-threatening complications with which gastric diverticulum can present, emergency physicians should keep this entity on their differential diagnosis.^1^,^3^ Providers need to be alert to the possibility of GD causing symptoms ranging from minor reflux and pain to refractory gastroesophageal reflux disease, hematemesis, perforation, or malignancy. Patients may present for the first time with upper GI hemorrhage or perforation needing emergent surgery.^1^,^3^ Providers should be familiar with the diagnostic options and treatment regimens available to better care for patients presenting with known or unknown gastric diverticulum.
